# Seeing More to Treat Better: Ultra-High Frequency Ultrasound as a Decision-Shaping Tool in Radiotherapy for Head and Neck Non-Melanoma Skin Cancer in a Single-Institution Feasibility Study

**DOI:** 10.3390/cancers18071083

**Published:** 2026-03-26

**Authors:** Emma D’Ippolito, Anna Russo, Luca Marinelli, Vittorio Patanè, Federico Gagliardi, Vittorio Salvatore Menditti, Angelo Sangiovanni, Nicola Maria Tarantino, Valerio Nardone, Alfonso Reginelli

**Affiliations:** Preicision Medicine Department, University of Campania Luigi Vanvitelli, Piazza Miraglia 2, 80138 Naples, NA, Italy; emma.dippolito@policliniconapoli.it (E.D.); anna.russo@unicampania.it (A.R.); luca.marinelli@unicampania.it (L.M.); vittorio.patane@unicampania.it (V.P.); federico.gagliardi@studenti.unicampania.it (F.G.); vittoriosalvatore.menditti@studenti.unicampania.it (V.S.M.); angelo.sangiovanni@unicampania.it (A.S.); nicolamaria.tarantino@unicampania.it (N.M.T.); alfonso.reginelli@unicampania.it (A.R.)

**Keywords:** external beam radiotherapy, head and neck cancer, non-melanoma skin cancer, radiotherapy planning, ultra-high frequency ultrasound, workflow integration

## Abstract

**Background/Objectives**: Accurate target delineation is critical in radiotherapy for head and neck non-melanoma skin cancer (NMSC), where tumor depth and subclinical extension are often underestimated by clinical and dermoscopic assessment alone. While high frequency ultrasound has shown value in surface-based radiotherapy techniques, the role of ultra-high frequency ultrasound (UHFUS) within external beam radiotherapy (EBRT) workflows remains poorly defined. **Methods**: We conducted a single-institution observational feasibility study including all consecutive patients with head and neck NMSC treated with definitive or adjuvant radiotherapy between July 2022 and July 2023 using a structured multidisciplinary workflow integrating pre-treatment UHFUS. UHFUS was systematically performed prior to CT simulation and incorporated into radiotherapy planning. The primary endpoint was the impact of UHFUS on radiotherapy decision-making, predefined as modification of target delineation, treatment intent, or beam modality selection. Secondary endpoints included feasibility, early local control, and late toxicity (descriptive). **Results**: Thirty patients were included (median age 85 years; range 66–99). UHFUS influenced at least one decision endpoint in 13 patients (43.3%). In the definitive radiotherapy cohort (n = 18), UHFUS modified gross tumor volume delineation in eight patients (44.4%), with an increase in median GTV from 17.5 cm^3^ to 24.3 cm^3^. Among patients initially referred for adjuvant radiotherapy (n = 12), UHFUS identified macroscopic residual disease in two cases, leading to a change in treatment intent from adjuvant to definitive radiotherapy. UHFUS supported beam modality selection in three patients by enabling safe use of electron therapy for superficial lesions. After a median follow-up of 24 months (range 12–24), no local recurrences were observed. Late toxicity was limited to grade 1 cutaneous events. **Conclusions**: Integration of UHFUS into EBRT planning for head and neck NMSC is feasible and clinically informative. UHFUS acts as a decision-shaping tool, influencing target delineation, treatment intent, and modality selection within a multidisciplinary workflow. These findings support further prospective evaluation of UHFUS-guided radiotherapy planning to standardize decision algorithms and assess long-term clinical impact.

## Simple Summary

This manuscript presents a single-institution feasibility study evaluating the integration of ultra-high frequency ultrasound (UHFUS) into external beam radiotherapy (EBRT) planning for head and neck non-melanoma skin cancer. The study introduces a structured multidisciplinary workflow in which pre-treatment UHFUS systematically informs radiotherapy decision-making. The primary endpoint was the predefined impact of UHFUS on target delineation, treatment intent, and beam modality selection. UHFUS influenced at least one decision endpoint in 43% of patients. The work positions UHFUS as a decision-shaping tool within CT-based EBRT planning and provides a reproducible implementation framework for future prospective validation.

## 1. Introduction

Non-melanoma skin cancer (NMSC) represents the most common malignancy in fair-skinned populations worldwide, with a steadily increasing incidence driven by population aging and cumulative ultraviolet exposure. Basal cell carcinoma (BCC) and cutaneous squamous cell carcinoma (SCC) account for the vast majority of cases and frequently involve the head and neck region, where functional preservation and cosmetic outcomes are critical components of treatment success.

Surgery remains the standard of care for most NMSC; however, a substantial proportion of patients present with clinical scenarios that complicate or preclude surgical management. Tumors arising in anatomically complex or cosmetically sensitive areas, lesions involving previously treated or reconstructed skin, and patients who are elderly, frail, or medically unfit for surgery pose a dual challenge: achieving durable local control while minimizing functional impairment and late toxicity. In these settings, definitive radiotherapy (RT) represents a well-established curative alternative, and adjuvant RT is recommended in the presence of high-risk pathological features such as positive margins, perineural invasion, or advanced T stage [1,2,3]. In selected advanced or high-risk cases, multimodal approaches including concurrent radiotherapy with platinum-based chemotherapy or immunotherapy may also be considered, reflecting the increasing complexity of treatment decision-making in this setting [4].

In head and neck NMSC, the therapeutic ratio of radiotherapy is highly dependent on accurate target definition. While modern RT techniques allow precise dose delivery, target delineation for superficial skin tumors still relies predominantly on clinical inspection, palpation, and dermoscopic assessment. These approaches, although widely adopted, are inherently limited in their ability to define tumor depth and subclinical extension, particularly in the presence of ulceration, fibrosis, post-surgical changes, or previously irradiated skin [5,6,7]. Consequently, tumor thickness and lateral extent may be underestimated, potentially leading to marginal misses or inappropriate selection of treatment technique and beam energy.

In dermatologic imaging, the terms high frequency ultrasound (HFUS) and ultra-high frequency ultrasound (UHFUS) are often used interchangeably, although they refer to distinct technological regimes. HFUS typically operates in the 15–30 MHz range, providing adequate penetration for dermal and subcutaneous assessment, whereas UHFUS employs frequencies ≥50 MHz, enabling near-microscopic resolution of superficial structures such as the epidermis and papillary dermis at the expense of reduced penetration depth. In the present study, we specifically employed [70 MHz], thus aligning with the [HFUS/UHFUS] category according to current consensus. High frequency ultrasound (HFUS) has emerged as a valuable adjunct in the assessment of superficial skin tumors, demonstrating improved accuracy in measuring tumor depth and lateral margins compared with clinical evaluation alone. Its integration into electronic brachytherapy and image-guided superficial radiotherapy workflows has shown that ultrasound-based measurements frequently exceed clinical estimates and can directly influence prescription depth, field size, and energy selection [8,9,10,11]. More recently, ultra-high frequency ultrasound (UHFUS), operating at frequencies ≥70 MHz, has enabled even finer spatial resolution of epidermal and dermal structures, offering detailed visualization of superficial tumor architecture and its relationship with underlying anatomical interfaces. The recent literature published in 2025 has further highlighted the expanding role of high-resolution dermal ultrasound in non-surgical skin cancer workflows, while also underscoring the limited standardization and the lack of evidence for integration into CT-based external beam radiotherapy (EBRT) planning [12,13]. In this context, we use the term “decision-shaping tool” to denote an imaging modality that directly informs clinical management, including surgical planning, target delineation, or follow-up strategy. This differs from purely diagnostic tools, which aim to detect or characterize lesions, and from staging tools, which are primarily intended to define disease extent. UHFUS may act as a decision-shaping modality by refining estimates of tumor thickness, margins, and superficial spread, thereby impacting procedural and therapeutic choices.

Thus, the current gap is not simply whether ultrasound can visualize superficial skin tumors, but how UHFUS findings can be operationally translated into reproducible EBRT planning decisions. Despite growing evidence supporting the role of HFUS and UHFUS in dermatology-led and surface-based radiotherapy techniques, their systematic integration into external beam radiotherapy (EBRT) workflows using linear accelerators remains poorly defined. In particular, there is limited literature describing how UHFUS findings can be incorporated into CT-based planning to inform gross tumor volume (GTV) delineation, treatment intent, and modality selection (photons versus electrons) in head and neck NMSC. This represents a relevant gap, as EBRT is frequently employed in complex or advanced cases where precise depth assessment and tissue sparing are especially critical.

To address this unmet need, a structured multidisciplinary workflow incorporating UHFUS was implemented at the University of Campania “Luigi Vanvitelli” for patients with head and neck NMSC referred for definitive or adjuvant radiotherapy. Within this workflow, UHFUS was systematically used prior to CT simulation to characterize tumor extent, guide skin marking, and support planning decisions in conjunction with clinical and dermoscopic evaluation.

Accordingly, this study was designed to address three main questions: first, whether UHFUS can be feasibly incorporated into routine EBRT planning for head and neck NMSC; second, whether it modifies clinically relevant planning decisions, including target delineation, treatment intent, and beam modality selection; and third, whether such integration can be achieved within a standard multidisciplinary workflow. The aim of the present study is to describe the implementation of this UHFUS-integrated radiotherapy workflow and to evaluate its impact on target delineation, treatment intent, and modality selection in a single-institution experience. Secondary objectives include assessing the feasibility of routine UHFUS incorporation into clinical practice and reporting early oncological and toxicity outcomes in a descriptive, hypothesis-generating framework.

## 2. Materials and Methods

### 2.1. Study Design

This study was designed as a single-institution observational feasibility study aimed at evaluating the implementation and clinical impact of a structured radiotherapy workflow integrating UHFUS for head and neck non-melanoma skin cancer (NMSC).

All consecutive patients treated according to the UHFUS-integrated workflow between July 2022 and July 2023 at the University of Campania “Luigi Vanvitelli” were included. During the study period, UHFUS assessment was systematically incorporated into the radiotherapy planning process for all eligible patients, and no patients meeting inclusion criteria were treated without UHFUS integration.

The study was conducted in accordance with the Declaration of Helsinki and was approved by the local Institutional Review Board. Due to the observational nature of the study and the use of standard-of-care procedures, the requirement for specific informed consent was waived.

The analysis was conceived as hypothesis-generating, focusing on decision-making processes and workflow feasibility rather than on comparative oncological outcomes.

### 2.2. Patient Selection

Eligible patients met the following criteria such as age ≥18 years, histologically confirmed diagnosis of basal cell carcinoma (BCC) or cutaneous squamous cell carcinoma (SCC), primary tumor located in the head and neck region, clinical stage T1–T4, N0, M0 according to the AJCC/UICC 8th edition and indication for definitive or adjuvant radiotherapy as determined by a multidisciplinary tumor board (MTB).

Patients were considered candidates for definitive radiotherapy if they were deemed unsuitable for surgery due to medical comorbidities, advanced age, anatomical complexity, or patient refusal. Adjuvant radiotherapy was recommended in the presence of high-risk pathological features, including positive or close surgical margins, perineural invasion, or advanced pathological T stage. Clinical and dermoscopic assessment was performed prior to UHFUS and served as the baseline evaluation. UHFUS was subsequently integrated into the planning workflow. When UHFUS findings differed from baseline assessment, final target delineation and treatment decisions were resolved within the multidisciplinary team and implemented by the treating radiation oncologist.

### 2.3. Multidisciplinary Workflow

All cases were discussed within a dedicated multidisciplinary tumor board, including radiation oncologists, dermatologists, radiologists with expertise in skin ultrasound, and surgeons when appropriate.

The UHFUS-integrated workflow consisted of the following predefined steps:

1. Clinical and dermoscopic assessment;
2. Pre-treatment UHFUS evaluation;
3. UHFUS-guided skin marking;
4. CT simulation and treatment planning;
5. Radiotherapy delivery;
6. Clinical and UHFUS-supported follow-up.

**Figure 1:**
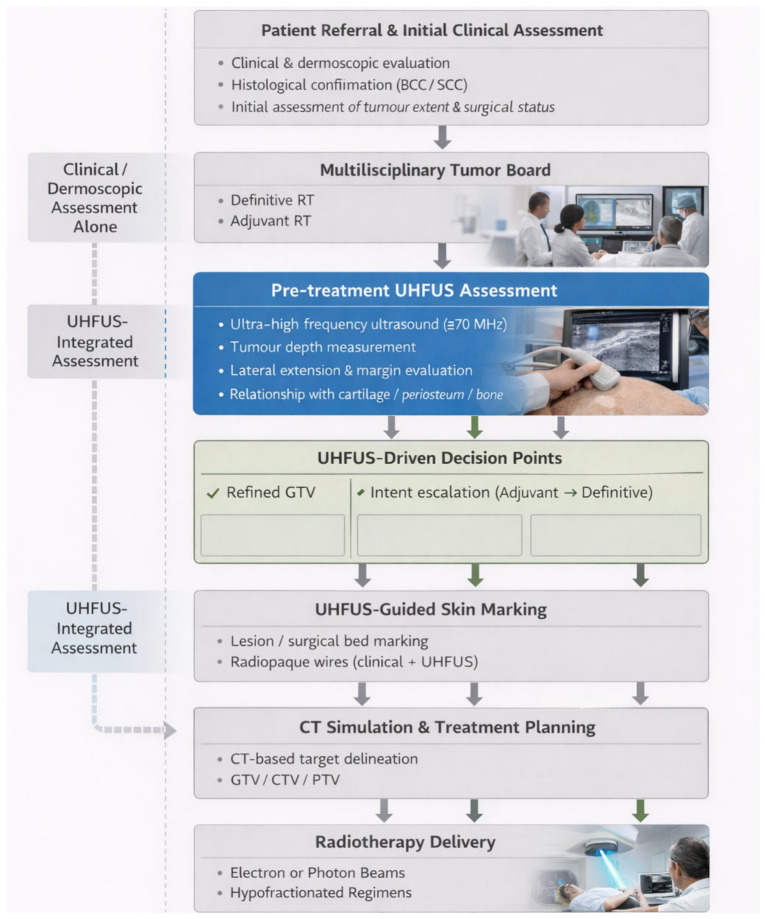
Structured ultra-high frequency ultrasound-integrated radiotherapy workflow.

UHFUS findings were formally reviewed during the planning phase and explicitly considered in radiotherapy decision-making. The UHFUS-integrated workflow is illustrated in Figure 1.

### 2.4. Ultra-High Frequency Ultrasound Protocol

Pre-treatment UHFUS was performed prior to CT simulation by a single experienced radiologist using a dedicated ultrasound platform (Vevo system, FUJIFILM VisualSonics, Amsterdam, The Netherlands) equipped with a 70 MHz linear probe.

To minimize probe-induced skin compression and ensure accurate depth assessment, a standoff technique was systematically employed. A thick layer of acoustic gel was applied to create a fluid interface between the transducer and the skin, avoiding direct pressure. In selected cases, a dedicated standoff pad was used to further optimize the distance between the probe and the epidermal surface. Particular attention was paid to maintaining the epidermis within the focal zone of the transducer, as compression artifacts are known to result in systematic underestimation of lesion thickness. All acquisitions were performed with minimal operator-applied pressure and standardized probe handling.

The examination systematically assessed:

- Maximum tumor depth, measured from the epidermal surface to the deepest hypoechoic tumor component;
- Lateral tumor extension;
- Echostructural characteristics and margin definition;
- Relationship with underlying anatomical structures, including cartilage, periosteum, and bone.

In ulcerated lesions, depth was measured from the estimated original epidermal surface based on surrounding intact skin. Measurements were obtained in at least two orthogonal planes, and representative images were stored in the institutional imaging archive.

For each patient, UHFUS findings were summarized in a structured report and made available to the radiation oncology team prior to target delineation.

### 2.5. Definition of Decision Impact

To ensure reproducibility and minimize post hoc interpretation, the impact of UHFUS on radiotherapy decision-making was predefined a priori and categorized as follows:

1. Target Delineation Impact
  o Any UHFUS-driven modification influencing the final gross tumor volume (GTV) delineation compared with clinical and dermoscopic assessment alone, without applying quantitative thresholds.
2. Treatment Intent Modification
  o Change from adjuvant to definitive radiotherapy following identification of macroscopic residual disease on UHFUS.
3. Treatment Modality Selection
  o Selection of photon versus electron beam therapy based on UHFUS-defined tumor depth and anatomical relationships.

Baseline clinical and dermoscopic assessment was performed before UHFUS evaluation. UHFUS findings were then reviewed during the planning phase by the treating radiation oncologist within the multidisciplinary framework, together with dermatological and radiological input. Final planning decisions were based on multidisciplinary consensus. Blinding was not applied, given the workflow-based nature of the study. We acknowledge that the pragmatic definition of decision impact, based on any UHFUS-driven modification influencing final planning, may introduce a degree of subjectivity. This approach was intentionally chosen to reflect real-world clinical workflows, but future studies should aim to define standardized quantitative thresholds for clinically meaningful modification. Macroscopic residual disease was suspected when UHFUS demonstrated a focal pseudonodular hypoechoic structure with irregular margins and subepidermal extension, rather than the linear or band-like echostructural changes more typically associated with postoperative scarring.

### 2.6. CT Simulation and Target Delineation

Patients were immobilized using a customized thermoplastic head mask and underwent planning CT (Aquilion One, Canon Medical Systems Corporation, Otawara, Japan) with a slice thickness of 3 mm.

Prior to simulation, the clinically and UHFUS-defined lesion (or surgical bed in the adjuvant setting) was marked on the skin using radiopaque wires, guided by UHFUS measurements and anatomical landmarks.

In the definitive setting,

- The GTV encompassed the clinically visible and UHFUS-defined tumor.
- A 5 mm isotropic margin was added to generate the clinical target volume (CTV).
- A further 3 mm margin was applied to define the planning target volume (PTV).
- In the adjuvant setting:
- The CTV corresponded to the surgical bed, incorporating UHFUS findings when applicable.
- A 3 mm margin was added to define the PTV.

Margin selection followed institutional standards and was not modified based on UHFUS findings, allowing improved target definition without adaptive margin reduction.

### 2.7. Radiotherapy Planning and Delivery

Radiotherapy was delivered using a linear accelerator (Elekta Infinity, Elekta AB, Stockholm, Sweden) with either megavoltage photon beams or electron beams.

Beam modality was selected according to tumor depth and anatomical relationships as defined by UHFUS:

- Electron beams were preferentially used for superficial lesions with limited depth and adequate separation from critical structures.
- Photon beams were selected for deeper lesions or anatomically complex sites.

Hypofractionated regimens were employed according to institutional practice and patient-related factors. All treatments were delivered on an outpatient basis. Hypofractionated regimens were employed according to institutional practice and selected on the basis of lesion size, anatomical site, treatment intent, and patient-related factors, including age, frailty, and performance status.

### 2.8. Follow-Up and Outcome Assessment

Patients were followed clinically every three months during the first year and every six months thereafter.

UHFUS was systematically used during follow-up to assess treatment response, detect subclinical recurrence, and document late cutaneous changes. Follow-up UHFUS findings were used for observational purposes only and did not trigger treatment modification within the scope of this analysis.

Toxicity was recorded according to the Common Terminology Criteria for Adverse Events (CTCAE), version 5.0.

The primary endpoint was the impact of UHFUS on radiotherapy decision-making, defined as any modification in target delineation, treatment intent, or modality selection.

Secondary endpoints included feasibility of routine UHFUS integration into the radiotherapy workflow, early local control (descriptive) and late cutaneous toxicity (descriptive). Clinical and dermoscopic assessment was performed before UHFUS and served as the baseline evaluation. The baseline clinical GTV was not formally “locked” in a blinded fashion, as the study was designed to reflect real-world workflow integration rather than an independent comparative contouring exercise.

### 2.9. Statistical Analysis

Data were analyzed using descriptive statistics. Continuous variables were reported as medians and ranges, and categorical variables as absolute numbers and percentages. No formal hypothesis testing was planned, in line with the exploratory nature of the study.

## 3. Results

Between July 2022 and July 2023, 30 consecutive patients with histologically confirmed non-melanoma skin cancer of the head and neck region were treated according to the UHFUS-integrated radiotherapy workflow and included in the analysis.

Patient, tumor, and treatment characteristics are summarized in Table 1. The median age was 85 years (range 66–99). Eighteen patients (60%) were male and twelve (40%) female. Most patients had an Eastern Cooperative Oncology Group (ECOG) performance status of 0–1 (60%), while 26.7% and 13.3% had ECOG 2 and 3, respectively.

Histology included 18 squamous cell carcinomas (60%) and 12 basal cell carcinomas (40%). Tumor stages according to AJCC 8th edition were heterogeneous, with T1–T2 disease in 70% of patients and locally advanced tumors (T3–T4) in 30%. All patients were clinically node-negative at presentation.

Radiotherapy was delivered with definitive intent in 18 patients (60%) and adjuvant intent in 12 patients (40%), based on multidisciplinary tumor board recommendations.

UHFUS influenced at least one predefined decision endpoint in 13 of 30 patients (43.3%). Decision impact categories were not mutually exclusive.

**Figure 2:**
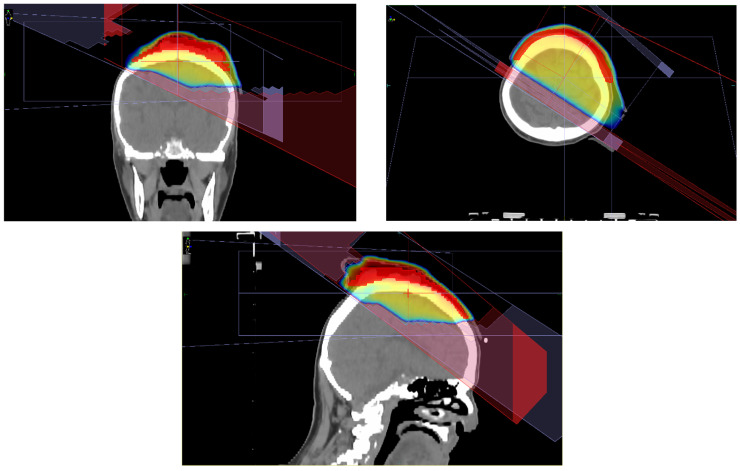
Representative ultra-high frequency ultrasound-driven modification of radiotherapy target delineation.

In the definitive radiotherapy cohort (n = 18), UHFUS resulted in a modification of gross tumor volume (GTV) delineation in eight patients (44.4%) compared with clinical and dermoscopic assessment alone. A representative case illustrating UHFUS-driven modification of target delineation is shown in Figure 2.

**Figure 3:**
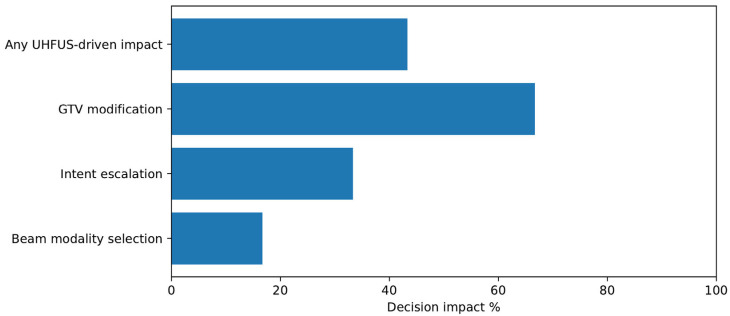
Impact of ultra-high frequency ultrasound on radiotherapy decision-making.

In these cases, UHFUS identified greater tumor depth and/or lateral extension than initially appreciated clinically, leading to expansion of the final GTV used for planning. The median GTV increased from 17.5 cm^3^ (clinical/dermoscopic assessment) to 24.3 cm^3^ after UHFUS integration. The overall UHFUS-driven decision impact is summarized in Table 2. The distribution of UHFUS-driven decision impact across predefined categories is shown in Figure 3.

Lesions exhibiting UHFUS-driven GTV modification commonly demonstrated hypoechoic nodular components with irregular margins and subepidermal extension that were not clearly delineable on surface inspection.

Among the 12 patients initially referred for adjuvant radiotherapy, UHFUS identified macroscopic residual disease in two cases (16.7%).

Both patients had squamous cell carcinoma (one scalp pT3 lesion and one nasal pT1 lesion) and had undergone R1 resection. UHFUS revealed pseudonodular hypoechoic formations with irregular margins and subepidermal extension, distinct from post-surgical tissue changes. In both cases, intent escalation from adjuvant to definitive radiotherapy was accompanied by the prescription of definitive-dose treatment.

Based on these findings, treatment intent was modified from adjuvant to definitive radiotherapy in both cases.

UHFUS influenced beam modality selection in three patients (10%).

In these cases, accurate assessment of limited tumor depth and clear separation from deeper critical structures allowed the safe selection of electron beam therapy instead of photon beams. Two patients had basal cell carcinoma of the scalp, and one had squamous cell carcinoma of the scalp (cT3).

This decision enabled preferential treatment of superficial target volumes while minimizing the dose to underlying bone and intracranial structures.

Radiotherapy was delivered using hypofractionated regimens in all patients. Sixteen patients (53.3%) received 55 Gy in 20 fractions, while fourteen patients (46.7%) received 60 Gy in 10 fractions, selected based on clinical indication and patient-related factors.

Electron beam therapy was used in 25 patients (83.3%), while five patients (16.7%) were treated with photon beams.

All treatments were completed as planned without interruption. Radiotherapy delivery characteristics and fractionation schedules are summarized in Table 3.

After a median follow-up of 24 months (range 12–24 months), no local recurrences were observed within or outside the treated volumes.

Late cutaneous toxicity was mild. The most common events were grade 1 telangiectasia in three patients and grade 1 hyperpigmentation in four patients. No grade ≥2 late skin toxicity was recorded.

Given the exploratory nature of the study and limited follow-up, oncological outcomes are reported descriptively.

This table summarizes the predefined categories of radiotherapy decision-making influenced by ultra-high frequency ultrasound (UHFUS), including modifications in target delineation, treatment intent, and beam modality selection. Decision impact categories are not mutually exclusive.

This table details the radiotherapy techniques and hypofractionated regimens employed in the study cohort, including beam modality and dose-fractionation schedules.

The structured multidisciplinary workflow illustrates the integration of ultra-high frequency ultrasound (UHFUS) into radiotherapy planning for head and neck non-melanoma skin cancer. Pre-treatment UHFUS assessment is systematically incorporated prior to CT simulation and informs predefined decision points, including target delineation refinement, treatment intent modification, and beam modality selection, within a CT-based external beam radiotherapy framework.

Representative case illustrating the impact of ultra-high frequency ultrasound (UHFUS) on radiotherapy target definition in head and neck non-melanoma skin cancer.

The bar chart illustrates the proportion of patients in whom ultra-high frequency ultrasound (UHFUS) influenced predefined radiotherapy decision endpoints. UHFUS-driven impact included modification of target delineation, treatment intent escalation, and beam modality selection. Decision impact categories are not mutually exclusive. Cutaneous events observed during follow-up extended beyond telangiectasia and hyperpigmentation and included hypopigmentation, fibrosis and induration, skin atrophy, focal dermal thickening, and scar-related architectural distortion. Occasional cases of post-inflammatory changes and vascular ectasia were also recorded. These findings were assessed clinically and, when applicable, correlated with ultrasound features such as altered echogenicity and dermal layer disruption.

## 4. Discussion

This study describes the implementation of a structured multidisciplinary workflow integrating UHFUS into radiotherapy planning for head and neck non-melanoma skin cancer and evaluates its impact on clinical decision-making in a real-world setting. The main finding is that systematic pre-treatment UHFUS assessment influenced radiotherapy decisions in more than 40% of patients, affecting target delineation, treatment intent, and modality selection. These results support the role of UHFUS as a decision-shaping tool, rather than a purely diagnostic adjunct, within EBRT workflows [14,15,16].

A relevant limitation of ultrasound-based assessment lies in the intrinsic geometric mismatch between 2D probe-derived imaging and 3D CT-based planning systems. This discrepancy may introduce spatial uncertainty in target delineation, particularly when extrapolating depth measurements to volumetric datasets. Potential strategies to mitigate this limitation include multiplanar acquisition with orthogonal scans, co-registration using anatomical landmarks, and the integration of ultrasound data into hybrid workflows. Emerging approaches, such as 3D ultrasound reconstruction and image fusion techniques, may further reduce geometric uncertainty and improve spatial consistency between modalities.

A key contribution of this work lies in the formalization of decision impact as the primary endpoint. Unlike previous reports in which ultrasound findings were described as narratively or implicitly linked to planning choices, we predefined and prospectively applied decision categories reflecting clinically meaningful actions [17]. This approach enhances transparency and reproducibility and allows the effect of UHFUS to be evaluated independently of oncological outcomes. In this context, the observation that nearly half of definitively treated patients experienced UHFUS-driven modification of GTV delineation underscores the limitations of clinical and dermoscopic assessment alone in defining tumor extent, particularly with respect to depth and subepidermal spread [18].

These findings are consistent with evidence from HFUS-guided electronic brachytherapy and image-guided superficial radiotherapy, where ultrasound frequently reveals tumor dimensions exceeding clinical estimates and directly informs prescription depth, field size, and energy selection [19,20,21]. However, most of this literature originates from dermatology-led, surface-based treatment paradigms. The present study extends these concepts into a CT-based EBRT environment, demonstrating that underestimation of tumor extent is not limited to superficial or orthovoltage techniques, but also affects linac-based radiotherapy when target definition relies predominantly on surface inspection.

Detailed quantitative comparisons of tumor depth and lateral extension were not systematically collected as standalone study endpoints, as the present analysis was designed to capture clinically relevant planning decisions rather than measurement-level correlations.

Beyond volumetric refinement, UHFUS had a clinically relevant impact in the postoperative setting. In two patients initially referred for adjuvant radiotherapy after R1 resection, UHFUS identified macroscopic residual disease, prompting a change from adjuvant to definitive treatment intent. This observation highlights a potential blind spot in current postoperative assessment pathways for head and neck NMSC. Surgical reports and pathology provide critical information, but they may not fully capture the residual disease burden in complex anatomical sites or in the presence of extensive post-surgical changes [22]. While our findings cannot support routine postoperative UHFUS in all patients, they suggest that targeted use in selected high-risk or ambiguous cases may improve treatment stratification and reduce the risk of undertreatment.

Although margins were intentionally not adapted in the present feasibility study, one potential future application of UHFUS may be to support margin refinement strategies once acquisition, interpretation, and decision thresholds are standardized. Another important aspect of our experience is the influence of UHFUS on treatment modality selection. In a subset of patients, accurate depth assessment and clear visualization of the interface with deeper structures allowed safe selection of electron beam therapy instead of photons [23,24,25]. This mirrors the depth-driven energy selection paradigm established in image-guided superficial radiotherapy programs and supports the concept of UHFUS as a gatekeeper for modality choice in EBRT. By enabling confident use of electrons in appropriately selected cases, UHFUS may facilitate more conformal dose delivery and improved sparing of underlying critical structures, particularly in anatomically sensitive head and neck sites [26,27,28].

Notably, margin selection in our workflow was intentionally conservative and not modified based on UHFUS findings. This design choice reflects a deliberate strategy to leverage improved target definition without introducing adaptive margin reduction, which could confound interpretation in a feasibility study. Although GTV expansion translated into corresponding target volume adaptation according to standard institutional margins, formal dosimetric analysis of organ-at-risk threshold changes was beyond the scope of this feasibility study. Importantly, no signal of clinically relevant excess late toxicity emerged in this small cohort. The absence of local recurrences and the low rate of late toxicity observed in our cohort, albeit with limited follow-up, suggest that improved delineation can be integrated safely into standard planning practices. Importantly, oncological outcomes were reported descriptively and should be interpreted as hypothesis-generating rather than confirmatory.

The multidisciplinary nature of the workflow represents another strength of this study. Systematic collaboration between radiation oncologists, dermatologists, and radiologists with expertise in skin ultrasound ensured that UHFUS findings were not interpreted in isolation but contextualized within clinical and dermoscopic assessment. This structured integration is particularly relevant in head and neck NMSC, where millimetric uncertainties can translate into meaningful differences in functional and cosmetic outcomes. Our experience supports the feasibility of incorporating UHFUS into routine clinical practice without disrupting standard radiotherapy workflows [29,30].

The limited sample size restricts the robustness and generalizability of our findings, and the observed proportion of UHFUS-driven decision impact should be interpreted cautiously within the exploratory nature of this feasibility cohort. Several limitations must be acknowledged. The limited sample size restricts the robustness and generalizability of our findings, and the observed proportion of UHFUS-driven decision impact should be interpreted cautiously within the exploratory nature of this feasibility cohort. Because UHFUS provides a high-resolution two-dimensional assessment acquired through direct probe contact, some degree of geometric uncertainty may arise when translating lesion extent into a three-dimensional CT-based planning system, particularly in the presence of skin compression or irregular surface anatomy. Histopathological correlation of UHFUS-derived tumor depth and lateral extension was not systematically available in this cohort. Therefore, the present study should not be interpreted as a diagnostic accuracy study, but rather as an implementation-focused analysis of decision-making impact. The single-institution design and limited sample size restrict generalizability, and the absence of a control cohort precludes comparative assessment of clinical outcomes. UHFUS examinations were performed by a single experienced operator, and inter-operator variability was not assessed. In addition, histopathological correlation of ultrasound findings was not systematically available, limiting conclusions regarding diagnostic accuracy. The absence of a comparison cohort treated without UHFUS limits the ability to determine whether similar planning modifications might have been achieved through conventional assessment alone. These limitations are inherent to the exploratory nature of the study and should be addressed in future prospective, multi-institutional investigations [31].

Scalability will depend on access to UHFUS equipment, availability of radiologists with dedicated expertise in skin ultrasound, and close multidisciplinary interaction. Therefore, immediate implementation may be more realistic in tertiary referral settings, while broader adoption will require standardized acquisition protocols and training. Despite these constraints, the present work provides a structured framework for integrating UHFUS into EBRT planning and offers a pragmatic template for other centers seeking to adopt this technology. Future studies should focus on standardizing UHFUS acquisition and reporting, defining quantitative thresholds for decision-making, and evaluating the impact of UHFUS-guided planning on long-term oncological and cosmetic outcomes.

## 5. Conclusions

In this single-institution feasibility study, the integration of ultra-high frequency ultrasound into the radiotherapy workflow for head and neck non-melanoma skin cancer proved to be practicable and clinically informative. Systematic pre-treatment UHFUS assessment influenced radiotherapy decision-making in a substantial proportion of patients, refining target delineation, modifying treatment intent in selected postoperative cases, and supporting modality selection in external beam radiotherapy.

These findings suggest that UHFUS represents a valuable decision-shaping tool when incorporated within a structured multidisciplinary framework, extending the benefits of ultrasound-guided assessment beyond dermatology-led and surface-based radiotherapy techniques into CT-based EBRT planning.

Although limited by its exploratory design and sample size, this experience provides a reproducible workflow and a rationale for the broader adoption of UHFUS in complex head and neck NMSC. Prospective multi-institutional studies are warranted to validate these observations, standardize UHFUS-guided decision algorithms, and define the impact of this approach on long-term oncological and cosmetic outcomes.

## Figures and Tables

**Table 1:** Patients, tumor and treatment characteristics.

Characteristics	N (%)
Age (years): median (range)	85 (66–99)
Male	18 (60%)
Female	12 (40%)
ECOG performance status	
0–1	18 (60%)
2	8 (26.66%)
3	4 (13.34%)
Histology	
SCC	18 (60%)
BCC	12 (40%)
Tumor stage (AJCC 8th)	
T1	9 (30%)
T2	12 (40%)
T3	8 (26.66%)
T4	1 (3.34%)
Nodal stage (AJCC 8th)	
N0	30 (100%)
Tumor site	
Forehead	5 (16.67%)
Scalp	4 (13.33%)
Superior cheek	6 (20%)
Inferior cheek	3 (10%)
Nose	8 (26.67%)
Lip	4 (13.33%)
Aim of radiation treatment	
Definitive	18 (60%)
Adjuvant	12 (40%)
Radiation type	
Photon beam	5 (16.67%)
Electron beam	25 (83.33%)

**Table 2:** Impact of ultra-high frequency ultrasound (UHFUS) on radiotherapy decision-making.

Decision Impact Category	Number of Patients	Percentage (%)
Any UHFUS-driven decision impact	13	43.3
Target delineation modification	8	26.7
Treatment intent modification	2	6.7
Modality selection (photons vs. electrons)	3	10.0

**Table 3:** Radiotherapy delivery and fractionation.

Parameter	Value
Electron beam therapy	25 (83.3%)
Photon beam therapy	5 (16.7%)
55 Gy/20 fractions	16 (53.3%)
60 Gy/10 fractions	14 (46.7%)

## Data Availability

Data can be made available on request.

## Abbreviations

The following abbreviations are used in this manuscript:

UHFUS	Ultra-high frequency ultrasound
NMSC	Non-melanoma skin cancer
BCC	Basal cell carcinoma
SCC	Squamous cell carcinoma
RT	Radiotherapy
EBRT	External beam radiotherapy
HFUS	High frequency ultrasound
MTB	Multidisciplinary tumor board
